# The Role of Regulatory Agencies in Agenda‐Setting Processes: Insights from the Italian Response to the COVID‐19 Infodemic

**DOI:** 10.1111/spsr.12465

**Published:** 2021-05-31

**Authors:** Fabrizio Di Mascio, Alessandro Natalini, Michele Barbieri, Donatella Selva

**Affiliations:** ^1^ University of Turin; ^2^ Parthenope University of Naples; ^3^ University of Tuscia

**Keywords:** Misinformation, Fake News, Social Media, Online Platforms, Disinformation

## Abstract

International organizations such as the WHO have worked to raise awareness of the massive infodemic that accompanied the COVID‐19 outbreak and made it hard for people to find trustworthy sources of information and reliable guidance for their decisions. Our contribution focuses on the Italian case, where the Communications Regulatory Authority (AGCOM) was able to act as first mover in its field so as to strategically frame the problem of disinformation in the absence of a pre‐existing policy intervention. An emerging body of research shows that the activity of formally independent regulators is not necessarily limited to the implementation of delegated regulatory competencies. We discuss the implications of the activity of independent regulators for the fight against disinformation during the COVID‐19 pandemic. We find that as a political actor in its own right, the Italian media regulator claimed control over sectoral expertise in order to shape the crucial first steps of the response to the infodemic.

## Zusammenfassung

Internationale Organisationen wie die WHO haben sich bemüht, das Bewusstsein für die massive «Infodemie» zu schärfen, die den COVID‐19‐Ausbruch begleitete und es den Menschen erschwerte, vertrauenswürdige Informationsquellen und verlässliche Entscheidungshilfen zu finden. Unser Beitrag untersucht den Fall Italiens, wo die Regulierungsbehörde für Kommunikation (AGCOM) als First Mover agieren und das Problem der Desinformation in Ermangelung einer bereits bestehenden politischen Intervention strategisch gestalten konnte. Die neuere Forschung zeigt, dass die Tätigkeit formal unabhängiger Regulierungsbehörden nicht notwendigerweise auf die Umsetzung delegierter Regulierungskompetenzen beschränkt ist. Wir diskutieren in diesem Beitrag die Auswirkungen der Tätigkeit unabhängiger Regulierungsbehörden auf den Kampf gegen Desinformation während der COVID‐19‐Pandemie. Wir stellen zudem fest, dass die italienische Medienregulierungsbehörde die Kontrolle über sektorales Fachwissen beanspruchte und so die entscheidenden ersten Schritte der politischen Antwort auf die Infodemie zu gestalten.

## Résumé

Des organisations internationales telles que l'OMS se sont efforcées de faire prendre conscience de l' « infodémie » massive qui a accompagné l'épidémie du COVID‐19 et qui a rendu difficile pour les gens de trouver des sources d'information dignes de confiance et des aides fiables à leurs décisions. Notre contribution se concentre sur le cas de l'Italie, où l'Autorité de régulation des communications (AGCOM) a été en mesure d'agir en tant que premier acteur dans son domaine afin d'encadrer stratégiquement le problème de la désinformation en l'absence d'une intervention politique préexistante. Des recherches récentes montrent que l'activité des régulateurs formellement indépendants ne se limite pas nécessairement à la mise en œuvre des compétences réglementaires déléguées. Nous examinons les implications de l'activité des régulateurs indépendants pour la lutte contre la désinformation pendant la pandémie COVID‐19. Nous constatons que le régulateur italien des médias a revendiqué le contrôle de l'expertise sectorielle afin de façonner les premières étapes cruciales de la réponse à l'infodémie.

## Introduction

Agenda‐setting is about getting policymakers to pay serious consideration to an issue (Green‐Pedersen and Walgrave 2014; Zahariadis 2016). Most scholars writing about crisis management note the potential agenda‐setting effects of crises. Political actors scan their environment for problems in order to promote their own preferred policy solutions, and they may seek appropriate crises for precisely that purpose (Kingdon 1995). Crises typically trigger exploitation games as actors seek to exploit them to defend and strengthen their positions and authority, to attract or deflect public attention, to protect established policies from pressures for change, or to discredit and dismantle the status quo. This implies that crises generate a contest between frames concerning their nature and severity, their causes, and the responsibility for their occurrence. Any theory of crisis exploitation needs to capture not just the emergence of frames, but also how actors bring an issue to the political agenda (Boin et al. 2009).

This research note analyzes the role of regulatory agencies in the COVID‐19 crisis exploitation game. It thereby aims to fill a gap in the emerging literature on the policy responses to the pandemic, which has so far neglected regulatory agencies when reviewing crisis management structures and processes (Boin et al. 2020). It also aims at bridging different areas of inquiry by making insights from crisis management literature available to research on the role of regulatory agencies in policy‐making. These two streams of literature share the focus on agenda‐setting in policy processes but have not yet engaged in a dialogue. More specifically, we analyze agenda‐setting dynamics in the sector of media regulation and focus on the issue of online disinformation. “Disinformation” can be defined as verifiably false or misleading information, created, presented and disseminated for economic gain or for intentionally deceive the public (Wardle and Derakhshan 2017). Indeed, the central role of social media platforms in the distribution and amplification of disinformation has recently justified initiatives to regulate internet platforms (Fukuyama and Grotto 2020).

The next section highlights that the light‐touch approach to the oversight of social media has been increasingly called into question over the past few years. The COVID‐19 crisis has provided further impetus for regulatory reform because the massive wave of disinformation that accompanied it has put the effectiveness of the response to the pandemic at risk (Hartley and Vu 2020). The following section presents our research framework. Finally, the empirical sections analyze how the Italian media regulator, the Authority of the Guarantees in the Communications (AGCOM), has exploited the pandemic to promote regulatory reform by attracting attention to the issue of disinformation.

We focus on Italy for a number of reasons. First, as the first Western country to be affected by COVID‐19, it was exposed to the highest level of uncertainty about the novel coronavirus. The Italian context was therefore favorable for disinformation. Second, Italy has conditions that facilitate an easier dissemination of and exposure to disinformation. Italy belongs to the cluster of polarized media systems that typically display high levels of societal polarization, populist political communication, and low levels of trust in media (Humprecht et al. 2020). Finally, Italy is one of a few countries in Europe where the media regulator has been delegated a wide set of monitoring and supervisory functions extending from telecommunications to audio‐visuals and publishing, with a view to ensure fair information and media pluralism since the mid‐1990s. As we discuss in the conclusive section, this institutional factor had direct consequences for agenda‐setting. The establishment of an independent media regulator ensured an issue like disinformation, that is directly related to AGCOM’s remit, an access point into the Italian political system.

## Regulatory Reform as a Response to the Infodemic

Given liberal democracies’ normative hostility to restrictions to speech, the regulation of social media platforms is commonly a sensitive and controversial topic in democratic systems (Rochefort 2020). The initial responses to disinformation have revolved around non‐regulatory interventions like the promotion of media literacy complemented by efforts to increase the visibility of authoritative content. However, the initial laissez‐faire approach to the regulation of social media platforms has not yielded the expected results, leading policy‐makers to increasingly call into question the libertarian view of the internet. Social media companies often advance this libertarian view when presenting themselves as neutral hosts of user content, meaning that they are not liable for what users do on a platform.

The laissez‐faire approach to the regulation of social media has become untenable after 2016 when large platforms, and especially Facebook, have come under sustained criticism for facilitating the circulation of disinformation in a series of critical elections in Europe and the United States. Following public revelations of foreign interference in the 2016 US presidential elections and the United Kingdom’s Brexit referendum, governments initiated a number of security actions that addressed disinformation as a threat to self‐determination and sovereignty. Disinformation campaigns can also undermine the integrity and fairness of the electoral process, as in the 2017 French presidential election which was followed by new electoral regulation in 2018. This regulation addressed false claims targeting candidates and required social media to disclose payments made to promote messages during elections (Tenove 2020).

Disinformation threats have also elicited responses at the level of the European Union. In early 2018 the European Commission established a High‐Level Expert Group (HLEG) on fake news and online disinformation, which recommended the adoption of self‐regulatory measures based on a clearly defined multi‐stakeholder engagement process, framed within a binding roadmap for implementation, and focused on a set of short and medium‐term actions (Saurwein and Spencer‐Smith 2020). Following these recommendations, in October 2018 the Commission introduced the EU Code of Practice on Disinformation, the first self‐regulatory set of standards to fight disinformation voluntarily signed by major platforms. However, the implementation of the Code showed major gaps in the accountability regime of social media platforms as it was difficult to assess the timeliness, completeness and impact of self‐regulatory actions as reported by the signatories (ERGA 2020). This evidence has stimulated the reflection on pertinent policy initiatives, including the introduction of more robust frameworks in which regulators would be made responsible to oversee the implementation of an enlarged set of commitments to fight disinformation to be made by online platforms. This reflection has become even more salient since mid‐February 2020, when the World Health Organization announced that the outbreak of the new coronavirus was accompanied by an ‘infodemic’. The use of this term captured the over‐abundance of information that makes it hard for people to find trustworthy sources and reliable guidance when they need it.

Awareness of disinformation peaked in the context of the infodemic when online dissemination of false claims became a major societal issue well beyond the realm of electoral politics. Part of the difficulty in addressing disinformation related to COVID‐19 is that the scientific consensus about the novel virus evolved constantly when new evidence became available. With social distancing measures in place, social media use has increased during the pandemic. Unlike electoral disinformation that had been at the center of the regulatory debate before the pandemic, COVID‐19 disinformation has the potential of threatening public health and of making efforts to achieve public acceptance of mitigation measures and vaccination even more challenging. When the pandemic continued to spread and its severe social and economic consequences became visible, disinformation increasingly exploited deep‐seated political and epistemological divisions in order to fuel the contestation of policy responses. To address disinformation related to COVID‐19, some social media platforms have introduced content moderation policies, raising a number of concerns about the discretionary nature of the development and application of these policies (Douek 2021). Our empirical section will show that this left room for media regulators who took advantage of the pandemic to call for a more robust framework to tackle disinformation.

## Research Framework

Over the last few decades, subsequent waves of administrative reforms have allowed regulatory agencies to become a distinct set of actors to which policymakers have delegated public authority across a wide range of policy sectors. The worldwide diffusion of regulatory agencies has gradually stimulated research about the role of agencies in policy‐making processes. According to this strand of research, agencies are not only crucial to the implementation of the delegated regulatory competencies, but they also systematically impact the initial stages of the policy process and the agenda‐setting stage in particular (Maggetti 2009).

Agenda‐setting determines which issues are taken up for active policy‐making and which alternatives are considered in policy‐making processes. Three main factors together shape agenda‐setting processes: institutional frameworks, framing and focusing events (Princen 2017). Institutional frameworks affect agenda‐setting by shaping the opportunities for actors to raise their concerns in policy‐making processes. Framing captures the process that brings issues to the agenda: often, the same issue can be represented in multiple ways, each of which has different implications for political action. An important part of agenda‐setting therefore consists of framing contests between actors who seek to impose competing definitions of the same issue upon their key audiences. Focusing events often have an important impact at the agenda‐setting stage because the immediately obvious harms of sudden, unusual, and disruptive events can lead political actors to identify new problems or to pay greater attention to existing but dormant issues, potentially leading to a search for new solutions (Birkland 1998).

Policy entrepreneurship is important for bringing institutional frameworks, framing and focusing events together. Policy entrepreneurs are those actors who actively try to link problems and solutions when favorable political conditions exist (Kingdon 1995). Whereas the wider literature on political agendas has paid great attention to the role of political agency in agenda‐setting processes, there has been little theoretical emphasis on the role of regulatory agencies as policy entrepreneurs (Jabotinski and Cohen 2020). Although regulatory policy entrepreneurs have been acknowledged as a relevant object of study in the literature, only a few studies so far shed light on how regulatory agencies can strategically push their preferences on political agendas (Littoz‐Monnet 2014).

We build on the work of Guaschino (2019) who conceptualizes the role of regulatory agencies in framing public problems as composed of four dimensions. We adapt this conceptualization to the study of agenda‐setting strategies that is based on the two key challenges for policy entrepreneurs: gaining attention and building credibility (Princen 2011). The first two dimensions of our framework – initiative and cognitive – pertain to the challenge of ‘gaining attention’ in agenda setting‐processes. The initiative dimension refers to the notion of regulatory agencies as ‘first movers’ that intervene in new issues by focusing on the technicalities of their intervention. It has been proposed that the formulation of policy is heavily influenced by the role of ‘first movers’, who benefit from an advantage in defining policy problems (Littoz‐Monnet 2014). The cognitive dimension involves framing a given issue in the ‘right way’: policy actors who are most successful in their agenda‐setting strategies are able to frame their issue in a way that arouses interest. However, gaining attention from policymakers is not sufficient for regulatory reform. The second challenge that regulators face is to build sufficient credibility to deal with a given issue. The instrumental dimension aims to discover whether regulatory agencies develop indicators, make studies, or create networks of experts in order to build up sufficient capacity to deal with a new issue. Finally, the leadership dimension refers to the ability of regulatory agencies to claim that a given issue is linked to a policy area over which they have authority.

Drawing on these four dimensions, in the next empirical section we apply a within‐case study design to the agenda‐setting dynamics of the regulation of disinformation in Italy. This approach is well‐suited for tracing how a focusing event like the pandemic has been exploited by the Italian media regulator in order to promote its concerns about disinformation. By comparing pre‐ and post‐crisis dynamics in the same entity (Italy), our longitudinal perspective reveals key actor‐level variables while keeping variables related to the institutional framework constant. The empirical analysis is based on secondary literature and official documents issued by EU and Italian institutions. These sources have been triangulated with semi‐structured interviews with experts who are knowledgeable about the regulation of online platforms in Italy.

## Tackling Disinformation in Italy before COVID‐19

The Brexit referendum raised attention to the issue of disinformation in Italy. In July 2016 AGCOM issued resolution 309/16/CONS launching a survey on digital platforms and the information system, which delved into the issue of disinformation. The analysis carried out in the survey supported the cognitive dimension of agenda‐setting by highlighting that disinformation represents “a vast and multiform phenomenon in terms of characteristics, actors involved, underlying motivations, communication techniques used to design fake news, tools and technologies employed and resources invested” (AGCOM 2018: 44). Given the complexity of disinformation, the “preferred policy approach is the so‐called ‘know‐it‐first’ principle that is based on a deep understanding of phenomena as a precondition to meet the challenges arising from the evolution dynamics of the information markets and by the rise of pathological forms of disinformation” (AGCOM 2019a: 1‐2).

The complexity of disinformation also implies that this issue should be addressed by various and complementary measures. To reach effectiveness, these measures “should be agreed and coordinated by different actors across the online disinformation field, thus requiring the involvement of all the stakeholders” (AGCOM 2018: 45). As a result, in November 2017 AGCOM established a Technical Roundtable for safeguarding media pluralism and fairness in the online platforms (resolution 423/17/CONS). The Roundtable was entrusted with the promotion of guidelines and codes of conduct complemented by other functions such as the monitoring of online disinformation and the promotion of initiatives for media literacy. It included representatives of the online platforms, of the most important national editors, of journalists, of the advertising industry, of consumers as well as academic institutions and research centres. In the framework of the Roundtable, ahead of the 2018 general elections AGCOM released the guidelines for the equal access to online platforms during the electoral campaign.

The collaborative approach enabled the regulator to collect data about disinformation in Italy that constituted the knowledge base for potential future regulation. With regard to the instrumental dimension of agenda‐setting, AGCOM carried out specific analyses, surveys and reports having as object the volume of disinformation, its contents and the patterns of information consumption. As for the volume of disinformation, the 2018 electoral campaign had a propulsive effect on the circulation of disinformation: the average impact of fake contents on the information contents of the national system increased from 1% (2% if we consider only the online contents) to about 6% (10% of online contents) in the period between August 2017 and August 2018 (AGCOM 2019a: 30). The results of the analysis raised even more concerns for media pluralism as the increase of disinformation was combined with the focus of fake content on news strictly connected to the political spheres (AGCOM 2019a: 36). Further, AGCOM applied big data analytics to dozens of millions of social media accounts to examine news consumption and the mechanisms of interaction of users through platforms. This study highlighted that the viralization of disinformation occurs in the framework of “echo chambers”, closed distinguished communities that result from the tendency of platform users to ideological polarization (AGCOM 2019a: 98).

The salience of disinformation was indeed closely linked to the extent to which this phenomenon has negative consequences for the formation of public opinion (AGCOM 2018: 5). In terms of the leadership dimension of agenda‐setting, AGCOM highlighted that its mission (i.e., the protection of media pluralism and fair information) inspired its monitoring of disinformation. The protection of such principles is “one of the funding objectives of the regulatory actions, which AGCOM pursues within its scope and mission” (AGCOM 2019a: 1). According to its institutional role, the action against disinformation falls under the regulator’s supervisory and monitoring activities of the wider media sector. AGCOM has also constructed the issue of disinformation as a field amenable to multilevel action, meaning that it has conducted most of its efforts within the EU framework that was perceived as increasing the chances of successful regulation of global actors such as online platforms. As revealed by the launch of the EU Code of Practice in 2018, AGCOM and the European commission shared an approach to disinformation in which multi‐stakeholder engagement is complemented by research on the impact of disinformation. AGCOM represented a precious source of expertise for EU‐level regulation since it led the task force that undertook the assessment of the implementation of the Code’s commitments by the signatories in the context of the monitoring activity of the European Regulators Group for Audio‐visual Media Services (ERGA 2020).

Between May 2017 and July 2019 AGCOM also engaged in cooperation with other Italian regulators to further specify its regulatory objectives. More specifically, a major survey of risks posed by big data was jointly conducted by AGCOM, the competition authority and the data protection authority. According to the policy recommendations that were developed in the context of the survey, national regulators should have been empowered with appropriate audit and inspection powers of algorithm profiling for information and content selection. In addition, they should have been vested with the power to assess the implementation of the internal rules adopted by digital platforms to cope with hate speech and disinformation (AGCOM 2019b: 4). It is worth noticing that the joint survey occurred in a context where political polarization prevented elected politicians to introduce any legislation. In September 2019 a proposal concerning the introduction of a parliamentary committee on fake news failed in the Chamber of Deputies due to staunch opposition by the center‐right parties.

## Tackling the Infodemic in Italy

AGCOM benefited from its “first mover” advantage in defining the disinformation issue in its own terms when it faced the infodemic. In particular, the media regulator benefited from the collaborative approach with the regulatees that it had promoted before the outburst of the health crisis. It set up a special roundtable “Digital Platforms and Big Data – COVID‐19 Emergency” in order to learn about the measures for combatting disinformation implemented by social media companies. In the framework of the roundtable, “the coordination was carried out by virtue of an action plan developed on the positive experience of cooperation with the stakeholders of the online information system” (AGCOM 2020a: 15). The roundtable was intended to act on several fronts, including “the monitoring of online disinformation in line with previous initiatives and consolidated methodologies, but with a specific focus on issues emerged with the Covid‐19 emergency” (AGCOM 2020a: 15). The production of quantitative analysis and monitoring data was also enhanced by the establishment of a data science task force on online disinformation.

With regard to the cognitive dimension of framing, AGCOM exploited the crisis in order to highlight that the perverse effects of disinformation were not confined to the electoral process. In the context of the COVID‐19 crisis, disinformation sources dealt with narratives focusing on conspiracy theories and issues capable of triggering irrational behaviors, such as, for instance, medical and pseudoscientific narratives about miracle cures and unreal remedies that put health in danger. According to AGCOM (2020a: 49), “in the specific case of the epidemic, where information quality is one of the key principles for contagion containment, the importance of preventing, promptly detecting and combating pathological disinformation phenomena once again emerges in all its evidence”. The information system already faced the critical issue of disinformation before the health emergency. It was precisely in the emergency that “disinformation showed its danger, directly affecting citizens’ safety and health” (AGCOM 2020a: 75). Further, online fraud and commercial disinformation did not go unnoticed during the epidemic. This implied that disinformation affects “citizens, their freedom of expression, but also the economy itself as it alters the information base on which customers and businesses build their consumption and production choices” (AGCOM 2020a: 75). This was extremely important in the context of the recovery phase, especially if taking into account the negative effects of disinformation on the reputation of Italian companies. In other words, “the ability to access quality information is of primary importance to the community. At all levels – from the medical‐healthcare one, to the ideological, political, social, and economic level – disinformation is able to generate distortions in business, institutions and citizens’ decisions, leading to failures of choices and policies” (AGCOM 2020a: 50). The disinformation problem and its consequences should be “considered a priority in a country where digital skills are very limited (AGCOM 2020a: 75).

Regarding the instrumental dimension of framing, the roundtable launched a special edition of the report on online disinformation dedicated to COVID‐19. The first three issues highlighted that citizens were at risk of relying on unqualified sources: in March 2020, in the midst of the emergency, more than 30% of internet users consulted websites containing disinformation, often accessing them through redirection from social media; comparing the first five months of 2020 with the same period of the previous year, the total volume of disinformation on any topic increased by 19%; disinformation sources devoted significant space to COVID‐19 related issues (around 40% of the total disinformation); articles on the epidemic disseminated by disinformation sources used a communication style based on terms leveraging negative emotions and anxiety (AGCOM 2020a: 46‐48).

With regard to the leadership dimension, the regulator highlighted that the monitoring activities on COVID‐19 disinformation was “linked to the exercise of the traditional regulatory, supervisory and control functions entrusted to AGCOM” (AGCOM 2020a: 5). The latter “has long since inaugurated a regulatory and analytical process in its expertise areas taking into account the entry of platforms into communication markets; moreover, it has explored innovative tools such as the use of technical roundtables and forms of self‐ and co‐regulation. However, there is a perceived difficulty in acquiring information and data from platforms, and, at the same time, the need to rapidly adapt the regulatory and legislative framework” (AGCOM 2020a: 78). According to the regulator, the increasing “platformization” of economies and societies poses serious and urgent matters, especially at a time of rapid digitalization as a result of the pandemic. In this context, “an efficient regulation of the platforms cannot disregard the fact that they are subject to obligations, making their activity more transparent and accountable to the Italian regulator” (AGCOM 2020a: 78).

AGCOM also benefited from a favorable European context that gave resonance to its arguments concerning the fight against disinformation. In May 2020 the ERGA report on disinformation acknowledged AGCOM’s scrutiny in the implementation of the EU Code. To improve the effectiveness of the EU Code, ERGA (2020) called for clear reporting obligations, more harmonized procedures and appropriate timeframes overseen by national regulators. However, the lack of homogeneity in legal systems applicable to social media platforms in different EU Member States implied that the revision of the EU regulatory framework should be accompanied by domestic reform. In Italy, domestic regulatory reform “would allow AGCOM to intervene earlier and more effectively to protect the right to information and pluralism of information” (AGCOM 2020a: 88). The regulator pointed out that “the development of monitoring systems is, in this sense, an unavoidable step towards the adaptation of existing regulations and the exercise of supervisory functions” (AGCOM 2020a: 88).

In June 2020, the President of the AGCOM recalled the need for an organic reform of the accountability framework applicable to platforms in the context of a parliamentary hearing on the reiterated proposal to set up a special inquiry committee on fake news. The agency leader welcomed the establishment of the committee in the context of a broader call for a “regulatory accountability” organic discipline of social media platforms. The President outlined the wide scope of the agency’s monitoring activities, including the investigation of disinformation patterns that was conducted in the context of ERGA’s scrutiny of social media platforms. The President also drew attention to the monitoring of COVID‐19 related disinformation patterns in order to highlight that the agency was best suited to implement audit and inspection powers in the oversight of online platforms (AGCOM 2020b).

AGCOM’s monitoring activity stood out in a context where elected politicians kept struggling to investigate disinformation. In early April 2020, a task force was established at the Italian Prime Minister Office and entrusted with the task of monitoring the spread of COVID‐19 disinformation. It was composed of representatives from the Civil Protection Department and the Ministry of Health as well as eight media experts. In June 2020, the task force released an operational program setting out the strategy and the priorities for the analysis of disinformation strategies. However, this program has not been followed by concrete measures to investigate disinformation. Instead, the Italian government focused its efforts on initiatives of institutional communication aimed at restoring the credibility of official information.

## Discussion and Conclusions

Pandemics require governments not only to consider the input of expertise in the decision‐making process, but also to ensure that evidence‐based crisis responses are effectively communicated to the public. In the context of the COVID‐19 crisis, this feature of public health crises has exposed the vulnerability of the governance to disinformation. Our analysis focused on the role that regulatory agencies play in agenda‐setting processes by raising attention to the deficiencies of self‐regulation by online platforms. Thereby, our research note highlights the contribution of political science to the understanding and management of disinformation as a key COVID‐19‐related policy issue.

While the debate about policy responses to the COVID‐19 crisis focuses on policy intervention by parliaments and governments, we shed light on agenda‐setting dynamics through which regulators intervene in crisis management, and claim that they have effective tools at their disposal in order to shape responses to the crisis. We drew on an emerging body of research in political science literature that shows how the involvement of regulators in policy‐making is not necessarily limited to the implementation of delegated regulatory tasks. Our study investigated the agenda‐setting process in Italy, a country characterized by a polarized media system and where parliament and government neglected the challenges of tackling disinformation prior to the COVID‐19 outbreak.

Our findings lead to three sets of consideration that resonate with previous studies of agenda‐setting. First, a focusing event like the pandemic had an important impact on the agenda setting process because it put the spotlight on the societal implications of disinformation that had hitherto received less attention (Birkland 1998). With regard to the cognitive dimension of agenda‐setting, the focus of AGCOM’s activity shifted from the impact of disinformation on political polarization during electoral campaigns to the implications of disinformation on the efficacy of the government’s health and economic policy responses to tackle the pandemic. Second, the Italian media regulator took advantage of its “first mover” status. Building on previous responses to disinformation, AGCOM made use of “multi‐stakeholder” arrangements (roundtable) and “evidence‐based” tools (indicators) that were deemed most suited to tackle the complexity of disinformation. Third, our analysis highlighted that institutional factors matter, because AGCOM’s institutional role had direct consequences on the leadership dimension of agenda‐setting. AGCOM acted as an institutional advocate of the regulation of disinformation because its monitoring activity of online platforms falls under its wider supervisory functions over the media sector. The inclusion in the multi‐level European regulatory network monitoring the implementation of the EU Code of Practice has provided AGCOM with further opportunities to participate in the policy‐making process.

To conclude, our note focused on the involvement of regulatory agencies across four dimensions of agenda‐setting processes as captured by previous studies (Guaschino 2019). The developments analyzed here show that regulators are able to act as political actors in their own right in the context of the COVID‐19 crisis. They do not confirm, however, that the regulators’ preferences have turned into enhanced regulation of online platforms. The COVID‐19 crisis is still unfolding and its developments have made apparent that the regulation of disinformation is a thorny issue: it raises the question of who controls public speech, triggering polarizing responses. As a next step, it would now be worthwhile to apply insights from the literature on the political role of regulators to the next stages of the COVID‐19 crisis and to track and compare how different trajectories of agenda‐setting processes influence policy‐making in different countries.^1^

## Data Availability

Data sharing is not applicable to this article as no new data were created or analyzed in this study.

## Footnotes

^1^
This article is the result of a common undertaking. However, the paragraphs “Research Framework” and “Discussion and Conclusions” can be directly attributed to Fabrizio Di Mascio; paragraph “Tackling the Infodemic in Italy” to Michele Barbieri; paragraphs “Introduction” and “Regulatory Reform as a Response to the Infodemic” to Alessandro Natalini; paragraph “Tackling Disinformation in Italy before COVID‐19” to Donatella Selva.
